# Distribution characteristics of circulating B cell subpopulations in patients with chronic kidney disease

**DOI:** 10.1038/s41598-023-47742-0

**Published:** 2023-11-27

**Authors:** Xuya Chen, Haoyang Guo, Danxia Jin, Yan Lu, Longyi Zhang

**Affiliations:** Clinical Laboratory, Affiliated Dongyang Hospital of Wenzhou Medical University, 60 West Wuning Road, Dongyang, 322100 Zhejiang China

**Keywords:** Immunology, Nephrology

## Abstract

This study compared the levels of circulating B cell subpopulations in patients with different stages of chronic kidney disease (CKD), investigated the effects of haemodialysis (HD) on the B cell-related immune spectrum in patients with end-stage renal disease, and evaluated the link between renal function and immune homeostasis. Overall, 197 patients with CKD (158 non-dialysis patients with CKD stages I–V and 39 end-stage patients undergoing maintenance HD) and 77 healthy controls were included. Compared to healthy controls, patients with CKD stages I–II showed no significant differences except for the proportion of transitional B cells; patients with CKD stage V showed a significant decrease in the proportions of transitional B cells and CD5^+^ B cells and a significant increase in double-negative (DN) B cells. Compared with early-stage patients with CKD, the absolute count of various B cell subpopulations in advanced-stage patients with CKD showed a significant decrease. The distribution of circulating B cell subpopulations in patients with CKD was significantly altered and was associated with CKD progression. Furthermore, the proportion of DN B cells and CD5^+^ B cells was inconsistent pre- and post-HD. This in-depth study of the immune status of patients with CKD may have important clinical value.

## Introduction

The incidence of chronic kidney disease (CKD) is 14.3% worldwide^1,2^. Of the 133 causes of death worldwide, CKD has risen in rank from 17th in 1990 to 12th in 2017^3^. In addition, CKD has been reported to severely affect overall survival^4^.

Regardless of whether immune suppression is caused by impaired immune cell function or immune activation is caused by the long-term stimulation of uraemic toxins, the coexistence of immune dysfunction and chronic inflammation is an important cause of complications in patients with CKD, including infection, malignant tumours, and cardiovascular diseases^5–8^. Abnormal immune function is critical in the development and progression of CKD. Previous studies on immune function have mostly focused on T cell immunity in patients with end-stage renal disease (ESRD). At present, little is known about the impact of CKD on the B cell-related immune system.

B lymphocytes are multifunctional cells that are a principal component of specific immunity. Generated by haematopoietic stem cells, they express diverse immunoglobulins and fight against a variety of pathogens^9^. B lymphocytes can be divided into different subgroups: transitional B cells, plasmablasts, naïve B cells, double-negative (DN) B cells, CD5^+^ B cells, and unswitched and switched memory B cells. Patients with advanced kidney disease have altered immune systems in comparison to healthier populations^10^. A few studies have shown a clear reduction in multiple B cell subsets in ESRD patients compared to those of controls^11–14^; however, these studies are limited to patients with ESRD. A recent study demonstrated that CD19^+^ B cell counts < 100 cells/μL may function as a novel biomarker of death in patients on haemodialysis (HD)^15^. Another study demonstrated that reduced B lymphocyte subsets were associated with poor survival in older patients with CKD^16^. Timely assessment of the immune status of B cells in patients with CKD can help identify prognostic indicators for follow-up monitoring of CKD and provide a theoretical basis for early intervention to improve prognosis.

These results indicate that B lymphocyte subpopulations may play different roles in the immune environment and prognosis of CKD. There is still no agreement on the distribution traits of B cell subpopulations in CKD development. Therefore, we analysed diverse subsets of B cells at distinct CKD stages to determine the immune characteristics of this population.

## Results

### Patient and demographic details

This study screened 248 patients with CKD and excluded 51 of them. Finally, a total of 197 patients were included in the analysis. Supplementary Figure S1 presents the study flowchart. The basic characteristics of the participants are presented in Table 1. The non-dialysis CKD group included 30 patients with CKD stage I, 30 patients with CKD stage II, 44 patients with CKD stage III, 12 patients with CKD stage IV, and 42 patients with CKD stage V. Moreover, 39 patients underwent maintenance HD. The non-dialysis CKD, maintenance HD, and healthy control groups did not significantly differ in age or sex. Compared with the healthy control group (205 [143–245]), the non-dialysis CKD group (162 [107–250]) and the maintenance HD group (70.8 [38.3–94.2]) showed a statistically significant decrease in the absolute count of B lymphocytes.

**Table 1:** Basic characteristics of the study population.

Characteristics	Non-dialysis CKD group (N = 158)	HD group (N = 39)	Healthy control group (N = 77)
Age (years)	55.5 [44–68]	55 [46–67]	56 [40–63]
Sex			
Male	83 (52.53%)	21 (53.85%)	32 (41.56%)
Female	75 (47.47%)	18 (46.15%)	45 (58.44%)
CKD stage			
I	30 (18.99%)		
II	30 (18.99%)		
III	44 (27.85%)		
IV	12 (7.59%)		
V	42 (26.58%)		
White blood cell count (× 10^9^/L)	6.1 [5.2–7.3]	5.5 [4.8–6.6]	6.1 [5.2–6.8]
Lymphocyte count (10^6^/L)	1.5 [1.2–2.0] ^ab^	1.0 [0.8–1.3]^a^	2.0 [1.8–2.4]
B Lymphocyte count (10^6^/L)	162 [107–250]^ab^	70.8 [38.3–94.2]^a^	205 [143–245]
UA (mmol/L)	400 [344–466]^a^	434 [382–493]^a^	299 [261–331]
BUN (mg/dL)	9.1 [5.9–20.6]^ab^	21 [18.1–23.6]^a^	5.2 [4.6–6.3]
Scr (mg/dL)	132 [84–344]^ab^	894 [721–1051]^a^	56 [51–69]
eGFR (mL/min)	47 [13–81]^ab^	4 [4–6]^a^	104 [97–118]

CKD, chronic kidney disease; UA, uric acid; BUN: blood urea nitrogen; Scr, serum creatinine; eGFR, estimated glomerular filtration rate.

Non-normally distributed data are represented by the median [interquartile spacing], and inter-group comparisons were conducted using the Mann–Whitney and Kruskal–Wallis tests, followed by Dunn's multiple comparison tests. Categorical variables are represented by the number of cases (percentage), and comparisons between groups were conducted using the chi-square test. Statistical significance for the *P* value was set at < 0.05 (compared with the Healthy Control group, ^a^*P* < 0.05; compared with the HD group, ^b^*P* < 0.05).

### B lymphocyte subsets in the non-dialysis CKD and healthy control groups

To further explore the role of B cell subpopulations in the progression of CKD, 158 non-dialysis patients with CKD were divided into three groups according to stage, and the levels of B cell subsets in the various CKD subgroups and in the healthy control group were analysed.

**Figure 1:**
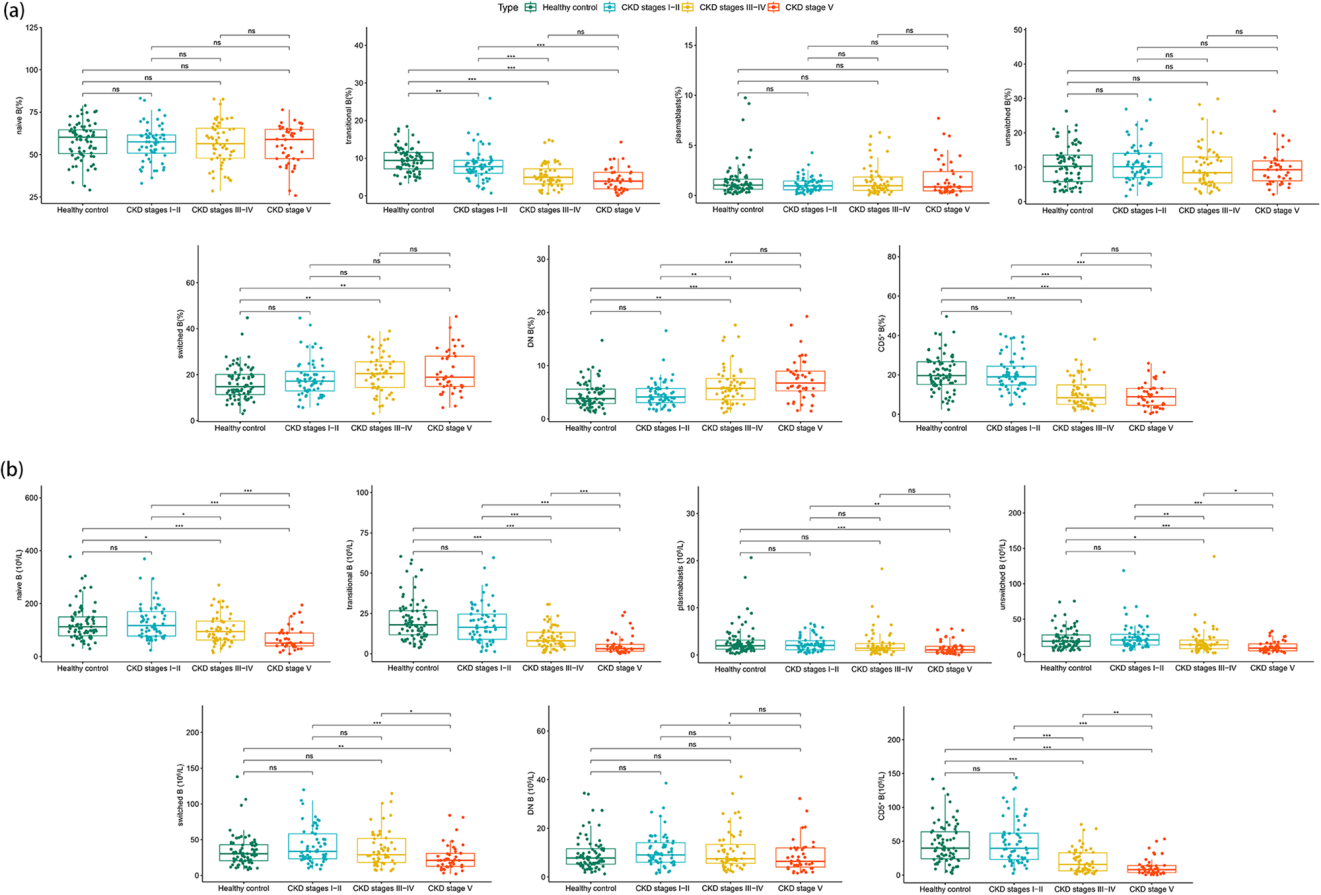
Differences in B lymphocyte subsets between the non-dialysis CKD group and the healthy control group. (**a**) Proportion of B cell subsets. (**b**) Absolute count of B cell subsets. Data with a normal distribution are compared between groups using independent sample t-tests. Data with a non-normal distribution are compared between groups using Mann–Whitney and Kruskal–Wallis tests, followed by Dunn's multiple comparison tests. The line inside the boxplot indicates the median. The top and bottom of the boxplot correspond to the 75th and 25th percentiles, respectively, and whiskers extend to 1.5 times the interquartile range. Statistical significance is set at a *P* value of < 0.05 (*P < 0.05, **P < 0.01, ***P < 0.001, ns = not significant).

Regarding the proportion of B cell subpopulations, as shown in Fig. 1a and Supplementary Table S1, there were no statistically significant differences in naïve B cells, plasmablasts, and unswitched B cells among the groups. Regarding the transitional B cells subgroup, the proportion of transitional B cells was significantly lower in patients with CKD stages I–II (7.78 [6.00–9.44]) than in the healthy control group (9.42 [7.18–11.6]. Moreover, there was a decreasing trend with CKD progression (CKD stages I–II: 7.78 [6.00–9.44]; CKD stages III–IV: 4.94 [3.16–7.19]; and CKD stage V: 3.92 [1.85–6.26]); however, the difference between CKD stage V and CKD stages III–IV was not significant. Regarding the DN B cells subgroup, the proportion of DN B cells in CKD stages III–IV (5.74 [3.59–7.60]) and CKD stage V (6.73 [5.26–8.96]) patients was significantly higher than that of healthy controls (3.82 [2.87–5.60]) and CKD stages I–II patients (4.12 [3.06–5.72]), showing an upward trend during CKD progression. In the CD5^+^ B cells subgroup, the proportion of CD5^+^ B cells in patients with CKD stages III–IV (8.37 [5.04–14.9]) and CKD stage V (8.89 [4.51–13.2]) was significantly lower compared to that of both healthy controls (19.6 [15.2–26.7]) and CKD stages I–II patients (19.0 [14.8–24.4]). Moreover, a statistically significant difference was observed in the proportion of switched B cells between CKD stages III–IV and CKD stage V relative to that of the healthy control group.

The absolute count of B cell subpopulations is shown in Fig. 1b and Supplementary Table S1. There was no statistically significant difference in the absolute count of each subgroup of B cells between CKD stages I–II and the healthy control group. As the disease progressed, the absolute count of each subgroup of B cells showed a downward trend. Patients with CKD stages III–IV exhibited statistically significant differences in naïve B cells, transitional B cells, unswitched B cells, and CD5^+^ B cells compared to those with CKD stages I–II. In particular, compared with patients with CKD stages I–II, the absolute count of various B cell subpopulations in CKD stage V patients significantly decreased: naïve B cells (117 [77.2–170] vs. 50.7 [40.1–88.7]), transitional B cells (16.4 [8.92–24.6] vs. 3.15 [1.55–5.81]), plasmablasts (2.01 [1.09–2.99] vs. 1.08 [0.56–1.84]), unswitched B cells (20.6 [13.4–28.5] vs. 9.10 [5.21–15.1]), switched B cells (33.6 [23.4–58.2] vs. 21.1 [12.8–30.9]), DN B cells (9.04 [6.06–14.1] vs. 6.41 [4.07–11.9]), and CD5^+^ B cells (39.5 [23.0–61.9] vs. 7.94 [3.78–14.0]). Statistically significant differences were observed between naïve B cells, transitional B cells, unswitched B cells, switched B cells, and CD5^+^ B cells when comparing CKD stages III–IV with CKD stage V.

### B lymphocyte subsets in the healthy control, non-dialysis CKD stage V, and HD groups

**Figure 2:**
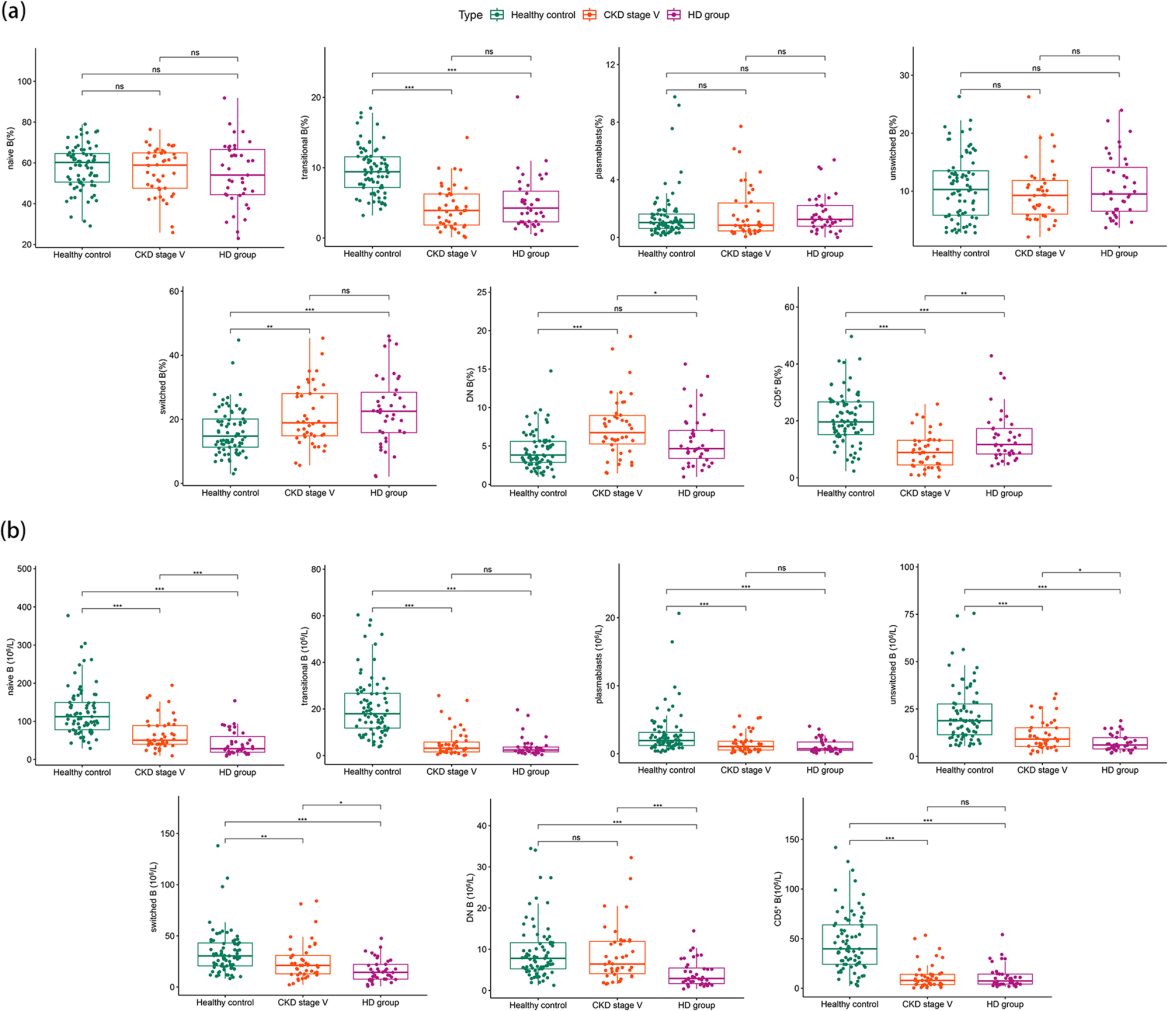
Differences in B lymphocyte subsets between the non-dialysis CKD stage V group, the HD group, and the healthy control group. (**a**) Proportion of B cell subsets. (**b**) Absolute count of B cell subsets. Data with a normal distribution were compared between groups using independent sample t-tests, while data with a non-normal distribution were analysed using Mann–Whitney and Kruskal–Wallis tests, followed by Dunn's multiple comparison tests. In the boxplot, the line inside indicates the median, while the top and bottom correspond to the 75th and 25th percentiles, respectively. The whiskers extend to 1.5 times the interquartile range. The threshold for statistical significance was set at *P < 0.05, **P < 0.01, ***P < 0.001; ns indicates not significant.

As shown in Fig. 2 and Supplementary Table S2, absolute counts of B cell subsets in patients undergoing HD were clearly lower than those of healthy controls. Compared to non-dialysis patients with CKD stage V, the proportion of DN B cells (6.73 [5.26–8.96] vs. 4.65 [3.38–7.03]) in patients undergoing HD decreased significantly, while that of CD5^+^ B cells (8.89 [4.51–13.2] vs. 11.7 [8.36–17.3]) increased significantly. The other subgroups were not statistically different. In addition, among patients undergoing HD, the absolute counts of naïve B cells (28.5 [19.1–60.2] vs. 50.7 [40.1–88.7]), DN B cells (2.91 [1.67–5.46] vs. 6.41 [4.07–11.9]), unswitched B cells (5.98 [3.90–9.84] vs. 9.10 [5.21–15.1]), and switched B cells (14.3 [7.68–22.1] vs. 21.1 [12.8–30.9]) were significantly lower than those in non-dialysis patients with CKD stage V.

### Correlation between B cell subsets and clinical parameters

The potential relationship between renal function indicators and different B cell subpopulations was analysed. Correlation analysis (Table 2) showed that the proportion and absolute count of transitional B cells and CD5^+^ B cells were negatively correlated with blood urea nitrogen (BUN), serum creatinine (Scr), and uric acid levels and positively correlated with glomerular filtration rate (eGFR). The proportion of DN B cells was negatively correlated with eGFR (r = − 0.340, *P* < 0.001) and positively correlated with BUN (r = 0.248, *P* = 0.002), Scr (r = 0.207, *P* = 0.009), and uric acid levels (r = 0.207, *P* = 0.009). Similarly, the absolute count of naïve B cells was positively correlated with eGFR and negatively correlated with BUN, Scr, and uric acid levels.

**Table 2:** Correlation analysis between B cell subsets and clinical parameters in the non-dialysis CKD group.

	UA	BUN	Creatinine	eGFR
Naïve B (%)	*r* = − 0.089	*r* = − 0.008	*r* = 0.018	*r* = 0.042
	*P* = 0.270	*P* = 0.916	*P* = 0.827	*P* = 0.602
Transitional B (%)	***r* = − 0.265**	***r* = − 0.347**	***r* = − 0.316**	***r* = 0.420**
	***P* = 0.001**	***P* < 0.001**	***P* < 0.001**	***P* < 0.001**
Plasmablasts (%)	*r* = 0.031	*r* = − 0.005	*r* = − 0.017	***r* = − 0.168**
	*P* = 0.701	*P* = 0.955	*P* = 0.833	***P* = 0.035**
Unswitched B (%)	*r* = 0.036	*r* = − 0.082	*r* = − 0.085	*r* = 0.077
	*P* = 0.656	*P* = 0.306	*P* = 0.291	*P* = 0.337
Switched B (%)	*r* = 0.142	*r* = 0.123	*r* = 0.093	*r* = − 0.138
	*P* = 0.077	*P* = 0.123	*P* = 0.246	*P* = 0.083
DN B (%)	***r* = 0.207**	***r* = 0.248**	***r* = 0.207**	***r* = − 0.340**
	***P* = 0.009**	***P* = 0.002**	***P* = 0.009**	***P* < 0.001**
CD5^+^ B (%)	***r* = − 0.341**	***r* = − 0.365**	***r* = − 0.328**	***r* = 0.525**
	***P* < 0.001**	***P* < 0.001**	***P* < 0.001**	***P* < 0.001**
Naïve B (10^6^/L)	***r* = − 0.239**	***r* = − 0.312**	***r* = − 0.277**	***r* = 0.398**
	***P* = 0.003**	***P* < 0.001**	***P* < 0.001**	***P* < 0.001**
Transitional B (10^6^/L)	***r* = − 0.303**	***r* = − 0.387**	***r* = − 0.350**	***r* = 0.516**
	***P* < 0.001**	***P* < 0.001**	***P* < 0.001**	***P* < 0.001**
Plasmablasts (10^6^/L)	*r* = − 0.088	***r* = − 0.183**	***r* = − 0.171**	*r* = 0.104
	*P* = 0.271	***P* = 0.022**	***P* = 0.031**	*P* = 0.192
Unswitched B (10^6^/L)	*r* = − 0.150	***r* = − 0.238**	***r* = − 0.220**	***r* = 0.257**
	*P* = 0.061	***P* = 0.003**	***P* = 0.005**	***P* = 0.001**
Switched B (10^6^/L)	*r* = − 0.098	***r* = − 0.206**	***r* = − 0.193**	***r* = 0.219**
	*P* = 0.220	***P* = 0.009**	***P* = 0.015**	***P* = 0.006**
DN B (10^6^/L)	*r* = 0.003	*r* = − 0.078	*r* = − 0.103	*r* = 0.061
	*P* = 0.971	*P* = 0.329	*P* = 0.200	*P* = 0.445
CD5^+^ B (10^6^/L)	***r* = − 0.305**	***r* = − 0.383**	***r* = − 0.339**	***r* = 0.547**
	***P* < 0.001**	***P* < 0.001**	***P* < 0.001**	***P* < 0.001**

UA, uric acid; BUN, blood urea nitrogen; Scr, serum creatinine; eGFR, estimated glomerular filtration rate; DN B, double-negative B cells.

Spearman's correlation was used to analyse the correlation between various B cell subsets and clinical data. A *P*-value < 0.05 was considered to indicate statistical significance.

Significant values are in bold.

## Discussion

In the present study, we observed significantly different distribution patterns of circulating B cells at various stages of CKD. Furthermore, by comparing the immunological features of patients with CKD pre- and post-HD, we assessed the implications of HD on circulating B cells.

Our study showed that, regardless of dialysis, the B lymphocyte subpopulations in patients with ESRD were significantly lower than those in healthy controls, consistent with the findings of prior studies^17^. Further analysis showed that compared with the healthy control group, the proportion of transitional B cells was significantly reduced in CKD stages I–II patients, while the changes in other B cell subsets were not significant. With the progress of CKD, the absolute count of various B cell subpopulations showed a decreasing trend. Moreover, the absolute numbers of various B cell subsets were significantly lower in patients with advanced CKD compared to early-stage patients with CKD. As expected, the levels of circulating B cells in patients with advanced CKD were not consistent with those in patients with early-stage CKD.

CD5^+^ B cells primarily produce IgM-type natural antibodies and play an essential part in tissue homeostasis, autoimmune diseases, anti-infection, and anti-atherosclerosis^18,19^. A previous study has reported a significant decrease in the number of CD5^+^ B cells in paediatric patients with chronic kidney failure^13^. In older patients with CKD, CD5^+^ B cells exhibit a significant inverse correlation with CKD development^20^. In this study, consistent with previous reports, CD5^+^ B cells were significantly reduced in non-dialysis CKD stages III–V and HD patients, both in terms of percentage and absolute count. Moreover, we found that the expression of CD5^+^ B cells in the early stages of CKD did not significantly differ from that in healthy controls and only exhibited a significant decrease as CKD progressed to stages III–V. In addition, the absolute count of CD5^+^ B cells showed statistical differences among various CKD subgroups.

DN B cells are rare in healthy individuals but are frequently represented in the older population and in patients with deforming arthritis and systemic lupus erythematosus (SLE)^21,22^. Recent research has suggested that DN B cells are linked to renal injury in SLE and serve as a marker for nephritis relief^23^. This implies a possible migration of DN B cells into relevant renal tissues during the course of the disease to produce antibodies that exert a pathogenic role. However, their clinical significance in CKD remains unclear. Our data showed that the DN B cell frequency was markedly raised in non-dialysis patients with CKD and exhibited a continuous upward trend as CKD progressed. In contrast, the absolute count of DN B cells declined during disease progression, and this decrease may be related to a decrease in the total number of B lymphocytes.

Transitional B cells (CD19^+^CD24^high^CD38^high^) are the most immature subtype of B cells in the blood, with the highest percentage and absolute count in children and significantly decreasing with age^24^. Our study showed that transitional B cells in patients with advanced CKD were significantly reduced. In the early CKD stages the proportion of transitional B cells begins to decrease and continues to gradually decrease as the disease progresses. Research has shown that the proportion of transitional B cells in patients with HD and kidney transplantation is lower than that in the healthy control group; this was not caused by immunosuppressants but rather due to the clinical manifestations of late-stage kidney disease^25^. Our research also confirms this point. No significant changes were observed in the distribution characteristics of transitional B cells in patients with advanced CKD, regardless of HD.

Transitional B cells are rich in B regulatory cells (Bregs) and are best described as B cells that primarily inhibit T cell responses by producing interleukin-10 (IL-10)^26,27^. A recent study found that kidney transplant recipients had a lower ratio of IL-10 to tumour necrosis factor-α, which is indicative of graft dysfunction, suggesting that transitional B cells or cytokines may be a new prognostic indicator for kidney transplantation^28^. Research has also shown that Bregs counts decline significantly in patients with ESRD and allergic purpura nephritis^29–31^. Therefore, exploring the role of transitional B cells and Bregs in the progression of CKD could help to identify prognostic indicators for follow-up monitoring of patients with CKD and could provide a theoretical basis for early intervention to improve CKD prognosis. Further research on this is warranted.

In this study, we discovered a substantial negative link between transitional B cells and CD5^+^ B cells and BUN, Scr, and Uric acid levels and a significant positive correlation between the proportion of DN B cells with renal function in patients with CKD. Previous research has revealed that the proportion of B cells in patients with different stages of CKD is negatively correlated with BUN, Scr, and cystatin C levels and positively correlated with eGFR^32^. Our research further strengthens these results and expands them to various B cell subsets. Notably, as CKD progresses, the DN B cells frequency gradually increases, whereas the transitional B cells frequency gradually decreases, suggesting that a low frequency of transitional B cells and a high frequency of DN B cells may be involved in renal function impairment in patients with CKD.

Furthermore, circulating B cell subpopulations exhibit age-related changes in humans: the number of naïve B cells and transitional B cells decreases, whilst that of depleted memory (IgD^-^CD27^-^) B cells increases with age^24,33^. Our data showed a similar ageing trend in circulating B cell subpopulations as CKD progresses. Interestingly, the rate and absolute counts of DN B cells were significantly reduced, while the frequency of CD5^+^ B cells was increased in the HD group compared with that of the pre-dialysis group. HD may partially restore the balance of B cells, thereby improving the immune function in patients with uraemia. However, this argument requires further investigation.

Our study presents limitations. First, the sample size was modest, and the follow-up period was brief. In addition, the implication of B cell subsets on mortality outcomes in patients with CKD was not studied. Second, immune regulation is a complex process, and the proportion and quantity of immune cells alone are insufficient to fully represent immune function. Thus, the relationship between the various phenotypes requires further clarification. Finally, further research is needed to analyse the relationship between differences in B cell subsets among patients with CKD at different stages and previously reported lower levels of IgG and subclasses^34^. Comprehensive and dynamic monitoring of immune function in patients with CKD may provide objective indicators for diagnosis, treatment, prevention, and prognosis of the disease.

In summary, patients with CKD exhibited significant alterations in B cell homeostasis compared with healthy controls. We observed an imbalance in the proportion of transitional B cells in the early stages of CKD, and patients with intermediate to advanced CKD exhibited severe B cell immunodeficiency. In particular, patients with CKD stage V showed a decrease in the frequency of transitional B cells and CD5^+^ B cells and an increase in the proportion of DN B cells. Absolute counts of various B cell subsets were negatively correlated with CKD progression. Moreover, HD may affect the expression of B cell subpopulations in ESRD patients.

## Methods

### Study population

This study included 158 patients with CKD not undergoing dialysis who were being treated at Dongyang Hospital, and affiliated with Wenzhou Medical University, 39 patients with ESRD treated with maintenance HD at a blood purification centre between January 2021 and January 2023. Using the Chronic Kidney Disease Epidemiology Collaboration formula to estimate the eGFR, non-dialysis patients with CKD were categorised into CKD stages I–II, CKD stages III–IV, and CKD stage V groups according to the Kidney Disease: Improving Global Outputs (KDIGO) definition criteria for CKD. Patients with ESRD receiving maintenance HD were assigned to the HD group, and 77 healthy volunteers were assigned to the control group.

The inclusion criteria were as follows: (1) compliance with the 2012 KDIGO definition of CKD; (2) age ≥ 18 years; (3) HD patients having a dialysis time of > 3 months; and (4) patients with complete case information. The exclusion criteria were: (1) history of active viral infections; (2) concomitant malignant tumours or haematological diseases; (3) recent surgical history or history of infection other than viral infections (< 3 months); (4) history of connective tissue diseases and recent immunosuppressive therapy (< 3 months); (5) pregnant or lactating women; and (6) an unstable condition, including severe gastrointestinal bleeding, severe heart failure, and cerebral haemorrhage.

This study was approved by the Ethics Committee of Dongyang Hospital, affiliated with Wenzhou Medical University, and all participating patients provided written informed consent. The study was performed according to the guidelines of the Declaration of Helsinki.

### Data collection

The demographic data of patients, including age and sex, and haematology indicators, including white blood cell count, total lymphocyte count, blood creatinine level, and BUN level, were collected.

### Flow cytometry analysis

Fresh whole peripheral blood samples were obtained from patients and tested within 24 h of collection. A multicolour flow cytometry panel was designed using Navios® (Navios; Beckman Coulter, USA) for monoclonal fluorescent antibody labelling of circulating B cells (Supplementary Table S3). According to the kit directions, 100 μL of whole blood was added to each flow tube, followed by the addition of premixed antibodies. Finally, the patient's peripheral blood lymphocyte subpopulations were detected in a ten-colour flow cytometer. Version 2.0 of the Kaluza software (Beckman Coulter) was used to analyse the data. Supplementary Figure S2 displays the gate control scheme.

Regarding the flow cytometry panel, B lymphocytes were defined by CD19 or CD20 positivity. According to the expression of CD24/CD38, B cells were divided into transitional B cells (CD24^high^CD38^high^) and plasmablasts (CD24^-^CD38^high^). Additionally, in the non-transitional B cells/non-plasmablasts population, naïve B cells (IgD^+^CD27^-^), DN B cells (IgD^-^CD27^-^), and unswitched (IgD^+^CD27^+^) and switched (IgD^-^CD27^+^) memory B cells were identified based on CD27/IgD. CD5^+^ B cells are defined by CD19^+^ and CD5^+^.

### Statistical analysis

We determined the proportion of lymphocyte subpopulations using flow cytometry. A haematology analyser (XN-9000; Sysmex, Japan) was used to determine the total lymphocyte number. Absolute counts for each subset were calculated using the proportions and absolute numbers of lymphocytes.

Statistical analyses were conducted using IBM SPSS Statistics software (version 23.0), and visualisation graphics were obtained using R (version 4.1.0). Normally distributed data are represented by (mean ± standard deviation), and the independent sample t-test was used for inter-group comparisons. Non-normally distributed data are represented by the median [interquartile spacing], and inter-group comparisons were conducted using the Mann–Whitney and Kruskal–Wallis tests, followed by Dunn’s multiple comparison test. Categorical variables are represented by the number of cases (percentage), and comparisons between groups were conducted using the chi-square test. Spearman's correlation was used to analyse the correlation between various B cell subsets and clinical data. A *P*-value < 0.05 was considered to indicate statistical significance.

### Supplementary Information

Supplementary Information.

## Data Availability

All data generated or analysed during this study are included in this published article.
