# Supplementary material for: Distribution characteristics of circulating B cell subpopulations in patients with chronic kidney disease
Source: Sci Rep. 2023 Nov 27;13:20797. doi: 10.1038/s41598-023-47742-0 (PMC10682455; doi:10.1038/s41598-023-47742-0)
Supplement: Supplementary file 1 — Supplementary Information. [file 41598_2023_47742_MOESM1_ESM.docx]

Distribution characteristics of circulating B cell subpopulations in patients with chronic kidney disease

Xuya Chen, Haoyang Guo, Danxia Jin, Yan Lu, Longyi Zhang*

Clinical Laboratory, Affiliated Dongyang Hospital of Wenzhou Medical University, 60 West Wuning Road, Dongyang, 322100, Zhejiang, China.

*Corresponding Author

* E-mail: [happy_zhang1y@163.com](mailto:happy_zhang1y@163.com) (Longyi Zhang)

**Supplementary Table S1**

Levels of B lymphocyte subsets in the non-dialysis CKD and healthy control groups.

|  | Healthy Control | CKD stages I-II | CKD stages III-IV | CKD stage V |
| --- | --- | --- | --- | --- |
|  | *N=77* | *N=60* | *N=56* | *N=42* |
| naïve B (%) | 60.2 [50.6–64.6] | 57.5 [50.9–61.5] | 56.4 [47.9–65.5] | 58.9 [47.6–64.9] |
| transitional B (%) | 9.42 [7.18–11.6] | 7.78 [6.00–9.44]**^a^** | 4.94 [3.16–7.19]**^ab^** | 3.92 [1.85–6.26]**^ab^** |
| plasmablasts (%) | 1.03 [0.62–1.62] | 0.96 [0.56–1.43] | 0.96 [0.50–1.83] | 0.84 [0.45–2.38] |
| unswitched B (%) | 10.3 [5.84–13.5] | 10.1 [7.00–14.0] | 8.40 [5.38–13.0] | 9.26 [6.03–11.8] |
| switched B (%) | 14.8 [11.3–20.1] | 17.2 [12.9–21.4] | 20.4 [14.4–25.6]**^a^** | 18.9 [14.9–28.0]**^a^** |
| DN B (%) | 3.82 [2.87–5.60] | 4.12 [3.06–5.72] | 5.74 [3.59–7.60]**^ab^** | 6.73 [5.26–8.96]**^ab^** |
| CD5^+^ B (%) | 19.6 [15.2–26.7] | 19.0 [14.8–24.4] | 8.37 [5.04–14.9]**^ab^** | 8.89 [4.51–13.2]**^ab^** |
| naïve B (10^6^/L) | 112 [78.0–150] | 117 [77.2–170] | 94.1 [62.2–134]**^ab^** | 50.7 [40.1–88.7]**^abc^** |
| transitional B (10^6^/L) | 18.0 [11.8–26.7] | 16.4 [8.92–24.6] | 8.06 [4.55–13.3]**^ab^** | 3.15 [1.55–5.81]**^abc^** |
| plasmablasts (10^6^/L) | 1.95 [1.22–3.15] | 2.01 [1.09–2.99] | 1.44 [0.95–2.44] | 1.08 [0.56–1.84]**^ab^** |
| unswitched B (10^6^/L) | 18.8 [11.4–27.7] | 20.6 [13.4–28.5] | 13.8 [8.42–20.5]**^ab^** | 9.10 [5.21–15.1]**^abc^** |
| switched B (10^6^/L) | 30.4 [20.6–43.0] | 33.6 [23.4–58.2] | 29.0 [18.2–51.6] | 21.1 [12.8–30.9]**^abc^** |
| DN B (10^6^/L) | 7.79 [5.24–11.6] | 9.04 [6.06–14.1] | 7.52 [5.62–13.4] | 6.41 [4.07–11.9]**^b^** |
| CD5^+^ B (10^6^/L) | 39.7 [24.1–63.9] | 39.5 [23.0–61.9] | 15.8 [6.33–32.9]**^ab^** | 7.94 [3.78–14.0]**^abc^** |

CKD, chronic kidney disease; DN B, double-negative B cells

Non-normally distributed data are represented by the median [interquartile spacing], and inter-group comparisons were conducted using the Mann-Whitney and Kruskal-Wallis tests, followed by Dunn's multiple comparison tests. Statistical significance for *P* value was set at < 0.05 (compared with Healthy Control group, **^a^***P* <0.05; compared with CKD stages I-II group, **^b^***P* <0.05; compared with CKD stages III-IV group **^C^***P* <0.05).

**Supplementary Table S2**

Levels of B lymphocyte subsets in healthy controls, non-dialysis CKD stage V, and HD groups.

|  | CKD stage V | HD group | Healthy control |
| --- | --- | --- | --- |
|  | *N=42* | *N=39* | *N=77* |
| naïve B (%) | 58.9 [47.6–64.9] | 54.0 [44.4–66.6] | 60.2 [50.6–64.6] |
| transitional B (%) | 3.92 [1.85–6.26]**^a^** | 4.26 [2.30–6.66]**^a^** | 9.42 [7.18–11.6] |
| plasmablasts (%) | 0.84 [0.45–2.38] | 1.25 [0.78–2.21] | 1.03 [0.62–1.62] |
| unswitched B (%) | 9.26 [6.03–11.8] | 9.50 [6.53–14.1] | 10.3 [5.84–13.5] |
| switched B (%) | 18.9 [14.9–28.0]**^a^** | 22.5 [15.9–28.5]**^a^** | 14.8 [11.3–20.1] |
| DN B (%) | 6.73 [5.26–8.96]**^a^** | 4.65 [3.38–7.03]**^b^** | 3.82 [2.87–5.60] |
| CD5^+^ B (%) | 8.89 [4.51–13.2]**^a^** | 11.7 [8.36–17.3]**^ab^** | 19.6 [15.2–26.7] |
| naïve B (10^6^/L) | 50.7 [40.1–88.7]**^a^** | 28.5 [19.1–60.2]**^ab^** | 112 [78.0–150] |
| transitional B (10^6^/L) | 3.15 [1.55–5.81]**^a^** | 2.37 [1.59–3.66]**^a^** | 18.0 [11.8–26.7] |
| plasmablasts (10^6^/L) | 1.08 [0.56–1.84]**^a^** | 0.72 [0.49–1.73]**^a^** | 1.95 [1.22–3.15] |
| unswitched B (10^6^/L) | 9.10 [5.21–15.1]**^a^** | 5.98 [3.90–9.84]**^ab^** | 18.8 [11.4–27.7] |
| switched B (10^6^/L) | 21.1 [12.8–30.9]**^a^** | 14.3 [7.68–22.1]**^ab^** | 30.4 [20.6–43.0] |
| DN B (10^6^/L) | 6.41 [4.07–11.9] | 2.91 [1.67–5.46]**^ab^** | 7.79 [5.24–11.6] |
| CD5^+^ B (10^6^/L) | 7.94 [3.78–14.0]**^a^** | 7.39 [4.22–14.3]**^a^** | 39.7 [24.1–63.9] |

CKD, chronic kidney disease; DN B, double-negative B cells

Non-normally distributed data are represented by the median [interquartile spacing], and inter-group comparisons were conducted using the Mann-Whitney and Kruskal-Wallis tests, followed by Dunn's multiple comparison tests. Statistical significance for *P* value was set at < 0.05 (compared with the Healthy Control group, **^a^***P* <0.05; compared with the CKD stage V group, **^b^***P* <0.05).

**Supplementary Table S3**

Staining panel for monoclonal fluorescent antibody labelling of circulating B cells.

| Fluorochrome | B cell subsets panel | | |
| --- | --- | --- | --- |
|  | Marker | Clone | Source |
| FITC | CD20 | Clone B9E9 | Beckman Coulter |
| PE | IgD | clone IA6-2 | Beckman Coulter |
| ECD | CD3 | clone UCHT1 | Beckman Coulter |
| PE-Cy5.5 | CD24 | clone ALB9 | Beckman Coulter |
| PE-Cy7 | CD27 | clone 1A4CD27 | Beckman Coulter |
| APC | CD19 | clone J4.119 | Beckman Coulter |
| AA750 | CD5 | clone BL1a | Beckman Coulter |
| PB | CD38 | clone LS198-4-3 | Beckman Coulter |
| KRO | CD45 | clone J.33 | Beckman Coulter |

FITC, Fluorescein Isothiocyanate; PE, Phycoerythrin; ECD, Phycoerythrin-Texas; PE-Cy5.5, Phycoerythrin-Cyanin 5.5; PE-Cy7, Phycoerythrin-Cyanin 7; APC, Allophycocyanin; AA750, APC- Alexa Fluor 750; PB, Pacific Blue; KRO, Krome Orange


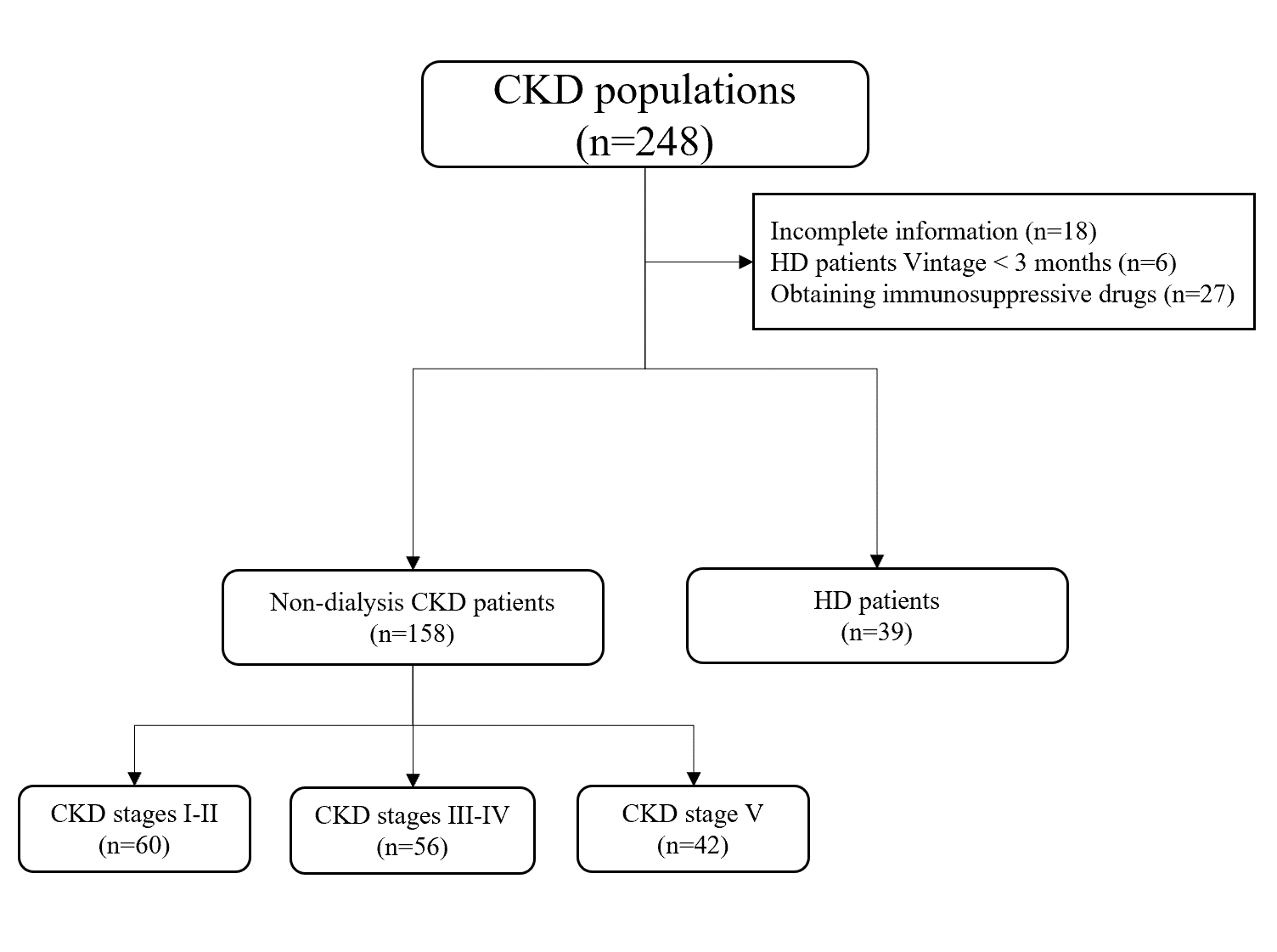


**Supplementary Figure S1**

Screening process for CKD patients to study the levels of circulating B lymphocytes. CKD: chronic kidney disease; HD: haemodialysis.


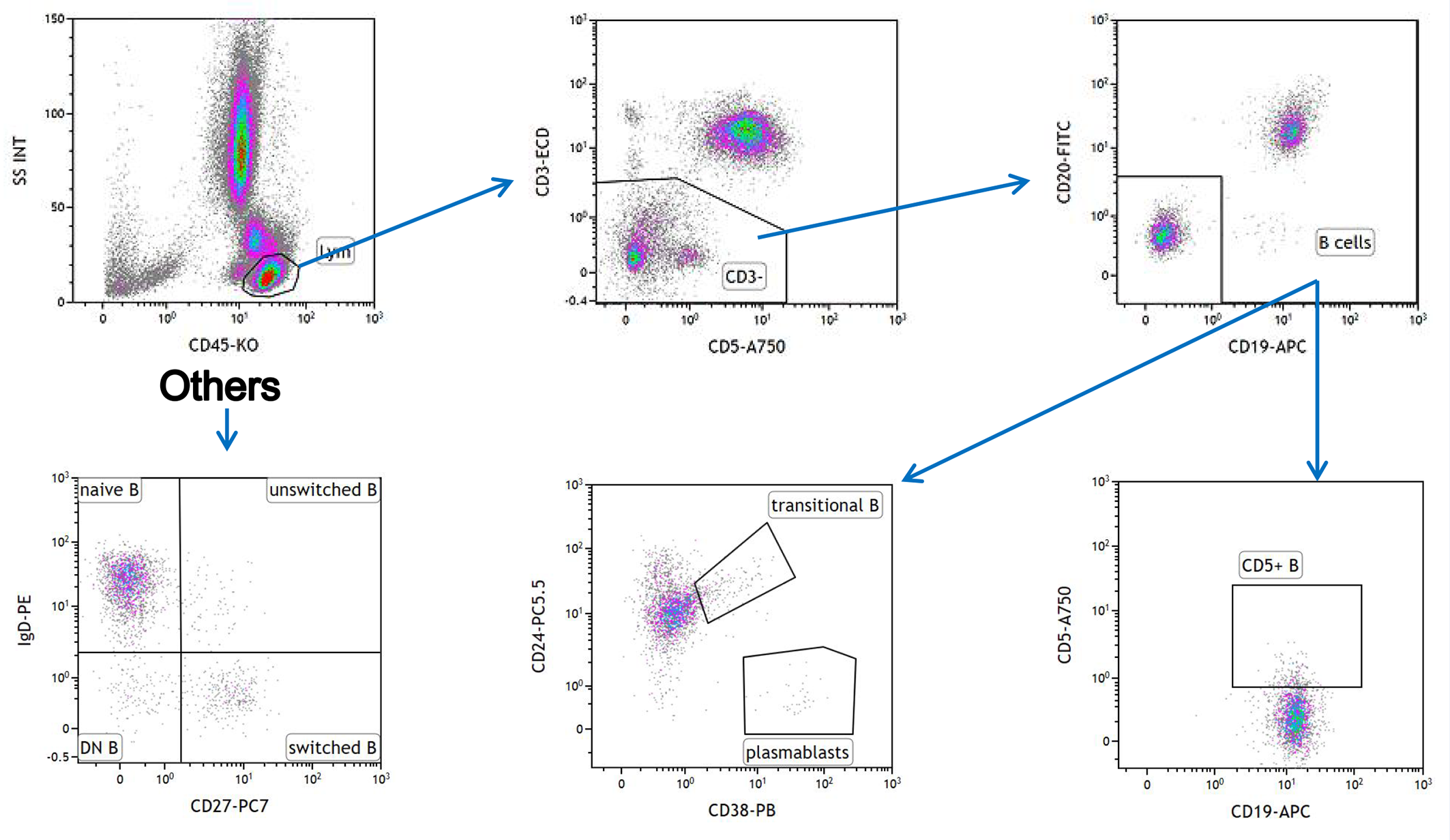


**Supplementary Figure S2**

Flow cytometry strategy for identifying circulating B lymphocytes.

Others= (“B cells” AND (NOT “transitional B cells”) AND (NOT “plasmablasts”))
